# Supplementary material for: Geometric and arithmetic characterization of 𝒟-module flatness with applications to tensor products
Source: PLoS One. 2025 Oct 16;20(10):e0334589. doi: 10.1371/journal.pone.0334589 (PMC12530596; doi:10.1371/journal.pone.0334589)
Supplement: Attachment — Submitted filename: Report.pdf [file pone.0334589.s001.pdf]

Report on

" Geometric and Arithmetic Characterization of D-Module Flatness with Applications to Tensor Products"

By Jian-Gang Tang, Huang-Rui Lei, Miao Liu, Jian-Ying Peng

The paper makes significant contributions to the theory of modules over rings of differential operators D-modules, addressing fundamental questions on flatness properties and tensor products across algebraic, geometric, and arithmetic contexts.

1. Geometric Characterization of Flatness:

The authors establish an equivalence between the flatness of D-modules and Lagrangian conditions on their characteristic varieties. This bridges symplectic geometry with homological algebra and resolves longstanding questions about the geometric interpretation of flatness, particularly for modules with irregular singularities.

2. Globalization Obstruction Theory:

A novel obstruction theory is developed to determine when locally defined flat modules extend to global flat modules. This theory leverages irregular Hodge filtrations and provides the first systematic framework for understanding the gap between local and global flatness in differential systems.

3. Arithmetic and Monoidal Applications:

The paper proves that in characteristic p, D-flatness is controlled by Frobenius semisimplicity and Lagrangian constraints on special fibers. Further, it demonstrates compatibility between tensor products, Beilinson-Bernstein localization, and irregularity-preserving integral transforms, with applications to geometric Langlands and mirror symmetry.

This manuscript meets PLOS ONE's criteria for originality, significance, and methodological rigor. The results will interest researchers in algebraic geometry, representation theory, and mathematical physics. I recommend acceptance pending minor revisions.

- Update references: Ensure all citations are current (e.g., include recent progress on irregular Hodge theory).
