# Supplementary material for: Geometric and arithmetic characterization of 𝒟-module flatness with applications to tensor products
Source: PLoS One. 2025 Oct 16;20(10):e0334589. doi: 10.1371/journal.pone.0334589 (PMC12530596; doi:10.1371/journal.pone.0334589)
Supplement: Attachment — Submitted filename: GAC.pdf [file pone.0334589.s002.pdf]

Review report on the paper

Geometric and Arithmetic Characterization of $\mathcal{D}$-Module Flatness with Applications to Tensor Products

by Jian-Gang Tang, Huang-Rui Lei, Miao Liu and Jian-Ying Peng

submitted to PLOS ONE

The authors of this paper aim to establish geometric and arithmetic characterizations of $\mathcal{D}$-module flatness, develop a framework for globalizing locally flat modules and uncover connections between tensor products, localization, and flatness in diverse mathematical contexts. Applications include compatibility theorems for Beilinson-Bernstein localization and arithmetic characterizations of flatness in characteristic p. The methods combine microlocal analysis, irregular Riemann-Hilbert correspondence, and p-adic techniques to yield new insights into the interplay between local and global properties of differential systems.

The results of the paper " Geometric and Arithmetic Characterization of $\mathcal{D}$-Module Flatness with Applications to Tensor Products", authored by Jian-Gang Tang, Huang-Rui Lei, Miao Liu and Jian-Ying Peng, are interesting.

There are some problems in this paper such as

- The proofs of the theorems in this paper, together with the entire exposition, must be reorganized into a formally rigorous and standard format.
- All mathematical symbols that appear in the paper must be given explicit definitions.
- References cited in the text should be carefully checked, with their sources specified in full detail and any omissions must be supplied. For example, the reference [11] on line 251 should be pinpointed to the exact theorem in that long paper, the reference [22] on line 252 should be specified to the precise page number or theorem in that book, and the missing citation on line 299 must be added.
- Lack of explanation for "m" in Definition 2.1.
- Every displayed equation in this paper is missing a punctuation mark "." at the end. It is recommended to add one to prevent readers from misunderstanding.
- It is suggested that the author provide the definition of symmetric monoidal structure.
- The clear definition of "$q-iso$" is not given in Definition 6.1 or the subsequent sections of the paper.
- The clear definition of "Ch^+" and "Tor" are not given in Theorem 6.2 or the subsequent sections of the paper.

- The clear definition of "$\mathbb{C}_x$" is not given in Theorem 7.3 or the subsequent sections of the paper.
- The clear definition of "$\mathcal{M} \otimes_{\mathcal{D}_X} (-)$" is not given in Definition 7.1 or the subsequent sections of the paper.
- The "microlocal characterization of irregularity" mentioned in Theorem 8.3 does not cite specific literature.
