# Supplementary material for: Geometric and arithmetic characterization of 𝒟-module flatness with applications to tensor products
Source: PLoS One. 2025 Oct 16;20(10):e0334589. doi: 10.1371/journal.pone.0334589 (PMC12530596; doi:10.1371/journal.pone.0334589)
Supplement: Attachment — Submitted filename: Response-to-Reviewer-2-comments-and-suggestions.pdf [file pone.0334589.s003.pdf]

Response to Reviewer 2

We sincerely thank the reviewer for their thorough reading of our manuscript and their positive assessment of our work. We are particularly grateful for their recognition of the geometric, homological, and arithmetic aspects of our study on D-module flatness and tensor products.

Below we provide a point-by-point response to the comments and suggestions:

Based on the comments from Reviewer 2, the following are specific revisions for the manuscript titled "Geometric and Arithmetic Characterization of \(\mathcal{D}\)-Module Flatness with Applications to Tensor Products." Each revision indicates the specific location in the original text (page number and approximate position) to ensure the paper meets the academic standards of PLOS ONE.

I. Paper Structure and Formatting Revisions

1. Add periods at the end of all displayed equations
   - Location: All displayed equations throughout the text
   - Revision: Add a period "." at the end of each displayed equation, for example:
     - Page 3, Definition 2.1: Add "." at the end of the equation
     - Page 4, Example 2.1: Add "." at the end of the equation
     - Page 5, Proposition 4.2: Add "." at the end of the equation
     - ... (and so on, consistently throughout the text)

II. Clarification of Symbols and Terminology

2. Define "m" in Definition 2.1
   - Location: Page 3, Definition 2.1
   - Revision: Add in the definition:
     > "where \( m \in \mathcal{M} \) denotes a local section of the module."

3. Clarify the term "q-iso"
   - Location: Page 7, Definition 6.1
   - Revision: Replace "q-iso" with "quasi-isomorphism" and add an explanation:
     > "where \( P_* \to \mathcal{M} \) and \( Q_* \to \mathcal{N} \) are \(\mathcal{D}_X\)-flat resolutions, and 'q-iso' denotes the quasi-isomorphism class in the derived category."

4. Define "$Ch^+$" and "Tor"
   - Location: Page 7, Theorem 6.2
   - Revision: Add before the theorem:
     > "Let \(\mathrm{Ch}^+(\mathrm{Mod}(\mathcal{D}_X))\) denote the

category of bounded below chain complexes of \(\mathcal{D}_X\)-modules, and \(\mathrm{Tor}_p^{\mathcal{D}_X}\) denote the derived functor of the tensor product."

5. Define "\(\mathbb{C}_x\)"
   - Location: Page 9, Theorem 7.3
   - Revision: Add at the end of the theorem:
     > "where \(\mathbb{C}_x\) denotes the skyscraper sheaf at the closed point \(x \in X\), i.e., the residue field of \(\mathcal{O}_{X,x}\)."

6. Define "\(\mathcal{M} \otimes_{\mathcal{D}_X} (-)\)"
   - Location: Page 8, Definition 7.1
   - Revision: Add in Definition 7.1:
     > "Here \(\mathcal{M} \otimes_{\mathcal{D}_X} (-)\) denotes the tensor product functor from left \(\mathcal{D}_X\)-modules to abelian groups."

7. Provide a clear definition or reference for "symmetric monoidal structure"
   - Location: Page 5, Proposition 4.2
   - Revision: Add before Proposition 4.2:
     > "Recall that a symmetric monoidal structure on a category consists of a tensor product functor, a unit object, and natural isomorphisms satisfying coherence conditions (see [5, Chapter 3] for details)."

III. Supplementation and Correction of References

8. Add missing citations (e.g., line 299)
   - Location: To be confirmed in the original text at "line 299"
   - Revision: Add a citation in the proof of Theorem 8.3:
     > "By the microlocal characterization of irregularity [7, Theorem 4.5], it suffices to show..."

9. Specify references [22]
   - Location: Page 13, Lemma 8.4
   - Revision:
     - Change "[22]" to: "[7, Thm 3.1.6]".

IV. Rigor in Proofs and Exposition

10. Reorganize the structure of theorem proofs
   - Location: Proofs of theorems throughout the text (e.g., Theorem 3.1, 7.3, 8.5, etc.)
   - Revision: Ensure each proof step is clear, consistently numbered, and logically coherent. Consider adding a "Step 1, Step 2, ..." structure where appropriate.

V. Other Details

11. Unify the use of symbols and terminology
   - Location: Throughout the text
   - Revision: Consistently use:
      - $\mathrm{Mod}(\mathcal{D}_X)$ instead of $\mathsf{Mod}(\mathcal{D}_X)$
      - $\mathrm{coh}(\mathcal{D}_X)$ instead of $\mathsf{coh}(\mathcal{D}_X)$
      - $\mathrm{Tor}$ instead of $\mathrm{Tot}$

12. Verify the accuracy of all cross-references
   - Location: Throughout the text
   - Revision: Ensure all references to "Theorem X.Y", "Proposition X.Y", etc., are accurate and without errors or omissions.

VI. Suggested Additional Content (Optional)

13. Briefly discuss the relationship of this work to recent developments in the introduction
   - Location: Page 3, end of the Introduction section
   - Revision: Add a sentence such as:
      > "Our work is also influenced by recent advances in $p$-adic Hodge theory [Ked] and irregular Hodge theory [Sab], which provide deeper insights into the arithmetic aspects of $\mathcal{D}$-modules."

Sincerely,
Jian-Gang Tang, on behalf of all authors
